# On a new species of *Amphilochus* from deep and cold Atlantic waters, with a note on the genus *Amphilochopsis* (Amphipoda, Gammaridea, Amphilochidae)

**DOI:** 10.3897/zookeys.731.19899

**Published:** 2018-01-23

**Authors:** Anne Helene S. Tandberg, Wim Vader

**Affiliations:** 1 University Museum of Bergen, Natural History Department, PO Box 7800, NO 5020 Bergen, Norway; 2 Tromsø University Museum, NO-9037 Tromsø, Norway

**Keywords:** *Amphilochus*, Amphipoda, BioIce, IceAGE, Mareano, new species, North Atlantic, taxonomy

## Abstract

*Amphilochus manudens* and *Amphilochopsis hamatus* are redescribed based on specimens from the BioIce, Mareano, and IceAGE programmes. The new species *Amphilochus anoculus* **sp. n.** is described based on material from the IceAGE programme and the preceding BioIce programme; it is separated from the closely related *Amphilochus manudens* by the absence of eyes, a symmetrically bilobed labrum, four setae on the maxilla 2 outer plate, a rounded corner of epimeral plate 3, and a robust seta at the tip of the telson. There are also clear differences in depth and temperature ranges. *Amphilochopsis hamatus* is shown to be closely related to *Amphilochus manudens* and *A. anoculus* and transferred to *Amphilochus* s. str.

## Introduction

The amphipod family Amphilochidae consists today of 15 genera, of which several are monotypic. There are ninety species, of which most are assigned to the possibly paraphyletic (Hoover and Bousfield 2001) genera *Gitanopsis* and *Amphilochus* (Horton et al. 2017). The family is cosmopolitan with the small genera seemingly restricted to specific geographic areas. Historically, the definition of Amphilochidae has been much like what Barnard and Karaman (1991) use as their diagnosis: ”Coxa 4 immensely broadened, coxae 2-4 with contiguous overlapping, not rabbeted, coxa 2 not hidden; coxa 1 very small and hidden by coxa 2. Peduncle of uropod 3 elongate. Telson entire, elongate.”

During the sorting of Amphilochidae material from the BioIce programme for a Master thesis in 2000, it became apparent that three groups of specimens had an anterodistal tooth on the propodus of pereopod 2. *Amphilochus manudens* and *Amphilochopsis hamatus* were already known from the literature (Sars 1890–95; Stephensen 1925; Gurjanova 1951), but the last group of specimens; with an anterodistal tooth and seemingly no eyes did not fit any of the described species. Specimens with the same morphological traits have since been found by the authors in amphipod material from Spitsbergen, the Faroe Islands, the Norwegian coast and in newly collected Icelandic material from the follow-up programme to BioIce: IceAGE (for information on IceAGE amphipod collections, see Brix et al. 2014; 2018). We therefore find it timely to describe a new species for the observed morphotype with the anterodistal tooth and no visible eyes. To be able to fully distinguish the new species from the known species it most resembles, morphological redescriptions of these are included, and the three species are genetically barcoded (COI-gene, Folmer et al. 1994) to show a clear separation of species both collected from Iceland (Jażdżewska et al. (2018)) and Norway (Boldsystems.org).

## Materials and methods

The material examined in this study comes from the programme BioIce in the years 1991–1997, the IceAGE-programme, and material in the collections of the University museums of Tromsø and Bergen, Norway. A few additional specimens derive from environmental monitoring studies around the Faroe Islands. For information on the collection of the material for BioIce, see Berge and Vader (1997), for the collection of IceAGE material, see Brix et al. (2014, 2018). Most of the new material at the University museum of Bergen comes from the Mareano programme; for collection of this material, see Buhl-Mortensen et al. (2015). The Amphilochidae-material from BioIce was sorted and described in Tromsø for an MSc-thesis (Tandberg 2000). Sample individuals were dissected using a binocular and mounted in rose-bengal-stained polyvinyl-lactophenol for examination under a light microscope. Pencil-drawings were made using a microscope fitted with a drawing tube; drawings were traced with ink and scanned. Digital inking on scanned hand-inked drawings followed procedures described by Coleman (2003, 2009). All scales on drawings are 0.1 mm unless otherwise stated.

Material from IceAGE and the collections from the University Museum of Bergen were identified and dissected for illustration of appendages using a Leica MZ12.5 stereo microscope. Temporary glycerine mounted and permanently mounted appendages (Faure medium) were drawn using a Leica 2500 compound microscope fitted with a camera lucida, and scanned pencil drawings were digitally inked in Adobe Illustrator following the method described by Coleman (2003, 2009). Animals used for COI-sequencing in Norway were photographed using a Leica DFC425 camera fitted with a motorised stacker on a Leica M205 binocular, and the Leica LAS 3.8 software for taking photos. Compilation of stacked photos into a single photo has been performed using Zerene Stacker 1.04 (setting P-max).

Further material for *Amphilochus anoculus* sp. n. comes from a survey in the Faroe-Shetland Channel (Mannvik et al. 2002), the Norwegian Sea and from the polar basin north of Spitsbergen (Tromsø Museum collections). Ecological data for *Amphilochus manudens* and *Amphilochopsis hamatus* were also gathered from the BioFar program (Nørrevang et al. 1994).

Sequencing of COI was performed through IceAGE (for details see Jażdżewska et al. 2018) and NorBOL (The Norwegian Barcode of Life, for details see Lörz et al. 2018).

BioIce material is held at the National Museum of Iceland, Reykjavik, Iceland (IINH-numbers).

IceAGE material is held at the Zoological Museum University of Hamburg, Centre of Natural History (CeNak), Germany (ZMH K-numbers).

NorAmph and other University of Bergen material is held at the University Museum of Bergen, Natural History Collections, Norway (ZMBN-numbers).

Material from University Museum of Tromsø is held at the Natural Collections University of Tromsø, Tromsø, Norway (TSZCr-numbers).

The material from the environmental studies performed by AkvaplanNIVA was kept for five years before it was destroyed: the identification of the amphipods of the survey was performed by the first author.

## Results

### Taxonomy

#### Order AMPHIPODA Latreille, 1816

##### Suborder GAMMARIDEA Latreille, 1802

###### Family AMPHILOCHIDAE Boeck, 1871

####### Genus *Amphilochus* Spence Bate, 1862

*Amphilochus* Spence Bate, 1862: 107; Stebbing 1906: 149; Barnard and Karaman 1991: 96

*Callimerus* Stebbing, 1876: 445

######## Amphilochus anoculus sp. n.

Taxon classificationAnimaliaAmphipodaAmphilochidae

http://zoobank.org/AD3ED2F5-F13B-4885-BD81-492F173B4EA1

######### Material examined.

from Icelandic (BioIce and IceAGE), Norwegian coastal and arctic (Svalbard) and Faroese waters. (For an extensive list of examined material see Table 1.).

**Table 1.** List of stations for examined species of *Amphilochus anoculus* sp. n., *A. manudens*, and *A. hamatus*. Asterisk * after museum-number indicates holo- and paratypes.

Species	Station name	Sampling programme	Collection number	Latitude (dec)	Longitude (dec)	Depth (m)	Temp (C)	BOLD-accension number	Note
*Amphilochus anoculus* sp. n.	BioIce 2087	BioIce		67,257, -17,446		735,0	-0,40		
	BioIce 2088	BioIce		67,239, -17,857		617,0	-0,40		
	BioIce 2094	BioIce		67,034, -17,570		303,0	1,70		
	BioIce 2100	BioIce		68,001, -19,421		1141,0	-0,60		
	BioIce 2107	BioIce		67,836, -19,555		905,0	-0,60		
	BioIce 2136	BioIce		66,726, -18,953		417,0	0,60		
	BioIce 2149	BioIce		66,749, -20,086		293,0	3,00		
	BioIce 2318	BioIce	IINH37886 (wet), IINH37916 (slide)	64,070, -9,030		996,0			
	BioIce 2325	BioIce		63,750, -10,183		555,0			
	BioIce 2367	BioIce	IINH37888, IINH37914*, IINH37915*	64,380, -9,430		719,0			Paratype (slides)
	3-1	Akvaplan NIVA Faroe project		60,348, -5,167		1088,0			
	8-1	Akvaplan NIVA Faroe project		60,591, -5,309		825,0			
	9-1	Akvaplan NIVA Faroe project		60,538, -5,206		921,0			
	13-2	Akvaplan NIVA Faroe project		60,483, -4,932		1022,0			
	15-1	Akvaplan NIVA Faroe project		60,553, -4,937		1055,0			
	15-3	Akvaplan NIVA Faroe project		60,553, -4,937		1055,0			
	81 03211	Tromsø Museum Collection tours	TSZCr 15516	63,167, 4,817		860,0			
	14968	Tromsø Museum Collection tours	TSZCr 14968	70,850, 15,383		2100,0			
	JM 369-05	UNIS AB321-2005	TSZCr 14338*	80,531, 10,578		819,0			Paratypes (wet)
	R405 RP59	Mareano	ZMBN_111537	72,140, 15,346		902,4	-0,41	AMPNB487-17	
	R479 RP156	Mareano		68,653, 10,301		2744,2	-0,82		
	R573 RP28	Mareano		70,872, 16,933		916,5	-0,64		
	R642 RP104	Mareano	ZMBN_104532	68,241, 9,243		2346,6	-0,84	AMPNB354-15	
	R653 RP108	Mareano		67,608, 8,392		1750,7	-0,84		
	R671 RP111	Mareano	ZMBN_104531	67,891, 9,875		777,2	-0,52	AMPNB353-15	
	R1180 RP86	Mareano		71,609, 32,992		304,9	2,84		
*Amphilochus anoculus* sp. n.	R1200 RP90	Mareano		70,854, 32,507		248,9	3,74		
	R1225 RP112	Mareano	ZMBN121953 *	70,475, 31,734		401,4	5,45		Paratype (slide)
	R1225 RP112	Mareano	ZMBN121959, ZMBN121960	70,475, 31,734		401,4	5,45		slides
	IceAGE 1010	IceAGE	ZMH K-47220	62,552, -20,395		1384,8			
	IceAGE 1010	IceAGE	ZMH K-47221	62,552, -20,395		1384,8			
	IceAGE 1054	IceAGE	ZMH K-47222	61,603, -31,377		2537,3			
	IceAGE 880	IceAGE	ZMBN121954	63,389, -8,157		686,0			
	IceAGE 880	IceAGE	ZMH K-47223	63,389, -8,157		686,0		AMPIV181-17	
	IceAGE 1010	IceAGE	ZMBN121955	62,552, -20,395		1384,8			
	IceAGE 1010	IceAGE	ZMH K-47224	62,552, -20,395		1384,8		AMPIV188-17	
	IceAGE 1057	IceAGE	ZMH K-47225*	61,642, -31,356		2504,7			Holotype (slide)
	IceAGE 1168	IceAGE	ZMH K-47226	67,606, -7,001		2372,6			
	IceAGE 1123	IceAGE	ZMH K-47227	67,214, -26,208		716,5			
	IceAGE 1172	IceAGE	ZMH K-47228	67,578, -6,935		2422,4			
	IceAGE 1181	IceAGE	ZMBN121956	67,658, -12,227		1827,0			
	IceAGE 1119	IceAGE	ZMBN121957	67,214, -26,242		696,9			
	IceAGE 871	IceAGE	ZMBN121958	62,737, -0,946		1577,4			
	IceAGE 1168	IceAGE	ZMH K-47229	67,606, -7,001		2372,6			
	IceAGE 1123	IceAGE	ZMH K-47230	67,214, -26,208		716,5			
	IceAGE 1172	IceAGE	ZMH K-47231	67,578, -6,935		2422,4			
	IceAGE 1172	IceAGE	ZMH K-47232	67,578, -6,935		2422,4		DNA-voucher: ZMH K-47232	
	IceAGE 1054	IceAGE	ZMH K-47233	61,603, -31,377		2537,3		DNA-voucher: ZMH K-47233	
	IceAGE 1159	IceAGE	ZMH K-47234	69,111, -9,917		2202,8			
	IceAGE 868	IceAGE	ZMH K-47235	62,152, 0,259		587,4			
	IceAGE 1123	IceAGE	ZMH K-47236	67,214, -26,208		716,5			
	IceAGE 1010	IceAGE	ZMH K-47237	62,552, -20,395		1384,8		DNA-voucher: ZMH K-47237	
	IceAGE 1006	IceAGE	ZMBN121952*	62,551, -20,375		1386,8			Paratype (slide)
*Amphilochus manudens* Spence Bate, 1862	BioIce 2096	BioIce		67,018, -17,578		300,0	1,70		
	BioIce 2207	BioIce	IINH37889	67,011, -22,596		81,0	8,30		
	BioIce 2213	BioIce		64,155, -23,971		260,0	7,00		
	BioIce 2215	BioIce	IINH37887	64,157, -24,261		213,0	6,90		
	BioIce 2221	BioIce		63,917, -25,273		240,0	6,50		
	BioIce 2236	BioIce		63,450, -24,680		293,0	6,90		
	BioIce 2237	BioIce	IINH37885	63,270, -24,408		293,0	6,90		
	BioIce 2273	BioIce		63,140, -24,983		313,0	7,00		
	BioIce 2288	BioIce		62,387, -22,677		1390,0	3,40		
	BioIce 2308	BioIce		63,250, -22,790		263,0	7,10		
	BioIce 2314	BioIce		63,703, -23,058		139,0	7,60		
	BioIce 2352	BioIce		63,783, -11,817		350,0			
	BioIce 2358	BioIce		63,167, -11,533		318,0			
	BioIce 2720	BioIce		64,430, -26,403		304,0	5,60		
	R405 RP59	Mareano		72,137, 15,341		899,6	-0,41		
	R423 RP69	Mareano		71,872, 17,142		355,3	5,53		
	R474 RP154	Mareano		71,073, 18,543		251,0	7,52		
	R503 RP51	Mareano		71,772, 25,975		321,1	4,42		
	R534 RP60	Mareano		70,675, 18,622		364,6	6,34		
	R608 RP87	Mareano		70,958, 21,120		149,0	6,57		
	R613 RP90	Mareano		70,769, 20,818		246,8	6,42		
	R618 RP91	Mareano		70,701, 21,025		258,8	6,38		
	R621 RP93	Mareano		70,673, 20,852		195,6	7,28		
	R631 RP99	Mareano		70,805, 19,702		178,6	6,87		
	R636 RP102	Mareano		70,622, 20,104		289,7	6,98		
	R657 RP109	Mareano		67,343, 8,638		849,6	-0,84		
	R721 RP126	Mareano		67,841, 11,809		183,2	6,87		
	R733 RP128	Mareano	ZMBN_94864	67,720, 10,272		219,2	7,34	AMPNB115-14	
	R754 RP132	Mareano		67,803, 9,685		823,5	-0,56		
	R786 RP10	Mareano		67,953, 9,589		1315,4	-0,84		
*Amphilochus manudens* Spence Bate, 1862	R782 RP11	Mareano		68,059, 9,468		1712,0	-0,81		
	R821 RP13	Mareano	ZMBN_104481	67,021, 8,223		556,0		AMPNB303-15	
	R870 RP19	Mareano		67,387, 11,622		128,3	6,29		
	R849 RP21	Mareano		67,401, 10,822		179,6	7,51		
	R1137 RP77	Mareano		72,574, 32,386		272,3	2,09		
	R1146 RP80	Mareano		72,103, 34,287		288,9	2,18		
	R1150 RP82	Mareano		72,093, 33,701		249,5	2,83		
	R1174 RP85	Mareano		71,618, 32,225		296,5	3,39		
	R1180 RP86	Mareano		71,609, 32,992		304,9	2,84		
	R1186 RP87	Mareano		71,421, 32,859		281,5	4,47		
	R1196 RP89	Mareano		71,187, 32,243		226,0	4,27		
	R1200 RP90	Mareano		70,854, 32,507		248,9	3,74		
	R1205 RP92	Mareano		70,574, 32,273		297,3	4,22		
	R1213 RP93	Mareano		70,771, 30,785		376,1	4,62		
	R1230 RP95	Mareano		70,117, 31,350		303,9			
	IceAGE 868	IceAGE	ZMH K-47238	62,152, 0,259		587,4			
	IceAGE 1082	IceAGE	ZMH K-47239	63,702, -26,394		724,4			
	IceAGE 1017	IceAGE	ZMH K-47240	62,931, -20,774		891,7			
	IceAGE 1032	IceAGE	ZMH K-47241	63,309, -23,158		289,4			
	IceAGE 878	IceAGE	ZMH K-47242	61,897, -10,230		781,4			
	IceAGE 868	IceAGE	ZMH K-47243	62,152, 0,259		587,4			
	IceAGE 1086	IceAGE	ZMH K-47244	63,709, -26,384		698,1			
	IceAGE 1219	IceAGE	ZMH K-47245	66,289, -12,347		579,1			
	IceAGE 1086	IceAGE	ZMH K-47246	63,709, -26,384		698,1			
	IceAGE 876	IceAGE	ZMH K-47247	60,406, -6,615		554,3			
	IceAGE 876	IceAGE	ZMH K-47248	60,406, -6,615		554,3		AMPIV183-17	
	IceAGE 878	IceAGE	ZMH K-47249	61,897, -10,230		781,4			
	IceAGE 878	IceAGE	ZMH K-47250	61,897, -10,230		781,4			
	IceAGE 878	IceAGE	ZMH K-47251	61,897, -10,230		781,4			
	IceAGE 1168	IceAGE	ZMH K-47252	67,606, -7,001		2372,6			
*Amphilochus manudens* Spence Bate, 1862	IceAGE 1104	IceAGE	ZMH K-47253	66,643, -24,533		118,8			
	IceAGE 1194	IceAGE	ZMH K-47254	67,078, -13,055		1573,5			
	IceAGE 1172	IceAGE	ZMH K-47255	67,578, -6,935		2422,4			
	IceAGE 867	IceAGE	ZMH K-47256	61,997, 0,507		302,5			
	IceAGE 866	IceAGE	ZMH K-47257	61,427, 1,351		169,1			
	IceAGE 870	IceAGE	ZMH K-47258	62,329, -0,102		1058,4			
	IceAGE 867	IceAGE	ZMH K-47259	61,997, 0,507		302,5			
	IceAGE 1123	IceAGE	ZMH K-47260	67,214, -26,208		716,5			
	IceAGE 1082	IceAGE	ZMH K-47261	63,702, -26,394		724,4		DNA-voucher: ZMH K-47261	
	IceAGE 866	IceAGE	ZMH K-47262	61,427, 1,351		169,1			
	IceAGE 867	IceAGE	ZMH K-47263	61,997, 0,507		302,5			
	IceAGE 868	IceAGE	ZMH K-47264	62,152, 0,259		587,4			
	IceAGE 1086	IceAGE	ZMH K-47265	63,709, -26,384		698,1		DNA-voucher: ZMH K-47265	
	IceAGE 867	IceAGE	ZMH K-47266	61,997, 0,507		302,5			
	IceAGE 867	IceAGE	ZMH K-47267	61,997, 0,507		302,5		DNA-voucher: ZMH K-47267	
*Amphilochus hamatus* (Stephensen, 1925)	BioIce 2077	BioIce	IINH37894	67,405, -17,104		1048,0	-0,50		
	BioIce 2087	BioIce		67,257, -17,446		735,0	-0,40		
	BioIce 2088	BioIce	IINH37892	67,239, -17,857		617,0	-0,40		
	BioIce 2090	BioIce	IINH37893	67,222, -17,816		539,0	-0,40		
	BioIce 2096	BioIce	IINH37891	67,018, -17,578		300,0	1,70		
	BioIce 2100	BioIce		68,001, -19,421		1141,0	-0,60		
	BioIce 2107	BioIce	IINH37890	67,836, -19,555		905,0	-0,60		
	BioIce 2136	BioIce	IINH37896	66,726, -18,953		417,0	0,60		
	BioIce 2149	BioIce		66,749, -20,086		293,0	3,00		
	BioIce 2213	BioIce	IINH37897	64,155, -23,971		260,0	7,00		
	BioIce 2236	BioIce	IINH37898	63,450, -24,680		293,0	6,90		
	BioIce 2237	BioIce		63,270, -24,408		293,0			
*Amphilochus hamatus* (Stephensen, 1925)	BioIce 2317	BioIce	IINH37889	64,117, -9,050		996,0			
	BioIce 2318	BioIce	IINH37900	64,070, -9,030		996,0			
	BioIce 2319	BioIce	IINH37901	64,017, -9,617		776,0			
	BioIce 2340	BioIce	IINH37902	62,133, -13,333		1302,0			
	BioIce 2367	BioIce	IINH37903	64,380, -9,430		719,0			
	BioIce 2410	BioIce	IINH37904	62,860, -21,735		1074,0	4,00		
	BioIce 2707	BioIce	IINH37905	63,922, -28,270		1407,0	3,70		
	BioIce 2719	BioIce	IINH37906	64,428, -26,403		300,0	5,60		
	R671 RP111	Mareano	ZMBN_104542	67,891, 9,875		777,2	-0,52	AMPNB364-15	
	R877 RP3	Mareano	ZMBN_104479	68,475, 9,785		2561,4	-0,80	AMPNB301-15	
	R776 RP4	Mareano		68,186, 10,354		799,9	-0,74		
	IceAGE 869	IceAGE	ZMH K-47268	62,270, 0,020		846,4			
	IceAGE 1006	IceAGE	ZMH K-47269	62,551, -20,375		1386,8			
	IceAGE 1006	IceAGE	ZMH K-47270	62,551, -20,375		1386,8			
	IceAGE 1019	IceAGE	ZMH K-47271	62,939, -20,744		913,6			
	IceAGE 1132	IceAGE	ZMH K-47272	67,641, -26,755		318,1			
	IceAGE 1172	IceAGE	ZMH K-47273	67,578, -6,935		2422,4			
	IceAGE 1123	IceAGE	ZMH K-47274	67,214, -26,208		716,5			
	IceAGE 1119	IceAGE	ZMH K-47275	67,214, -26,242		696,9			
	IceAGE 1119	IceAGE	ZMH K-47276	67,214, -26,242		696,9		DNA-voucher: ZMH K-47276	
	IceAGE 1010	IceAGE	ZMH K-47277	62,552, -20,395		1384,8			
	IceAGE 1010	IceAGE	ZMH K-47278	62,552, -20,395		1384,8		DNA-voucher: ZMH K-47278	
	IceAGE 1172	IceAGE	ZMH K-47279	67,578, -6,935		2422,4		DNA-voucher: ZMH K-47279	
	IceAGE 869	IceAGE	ZMH K-47280	62,270, 0,020		846,4			
	IceAGE 1010	IceAGE	ZMH K-47281	62,552, -20,395		1384,8			

Holotype: IceAGE ZMH K-47225, female 3 mm (slide).

Paratypes: Slides: BioIce 2367 male, 3 mm IINH37914; BioIce 2367 female, 3 mm IINH37915; MareanoR1225-RP112 female 4 mm ZMBN121953; IceAGE 1006 male, 3 mm ZMBN121952. Wet-sample: TSZCr 14338 (8 specimens).

######### Type locality.

 ZMH K-47225: IceAGE station 1057 (61.6417, -31.3562) (2504m).

######### Paratype localities.

 IINH37914, IINH37915: BioIce station 2367 (64.3800, -9.4300) (719m); TSZCr 14338: UNIS course-station JM 369-05 (80.5313, 10.5777) (819 m); ZMBN121953: Mareano station R1225-RP112 (70.4748, 31.7340) (401 m); ZMBN121952: IceAGE station 1006 (62.5508, -20.3750) (1386 m).

######### Distribution.

**Figure 1.**
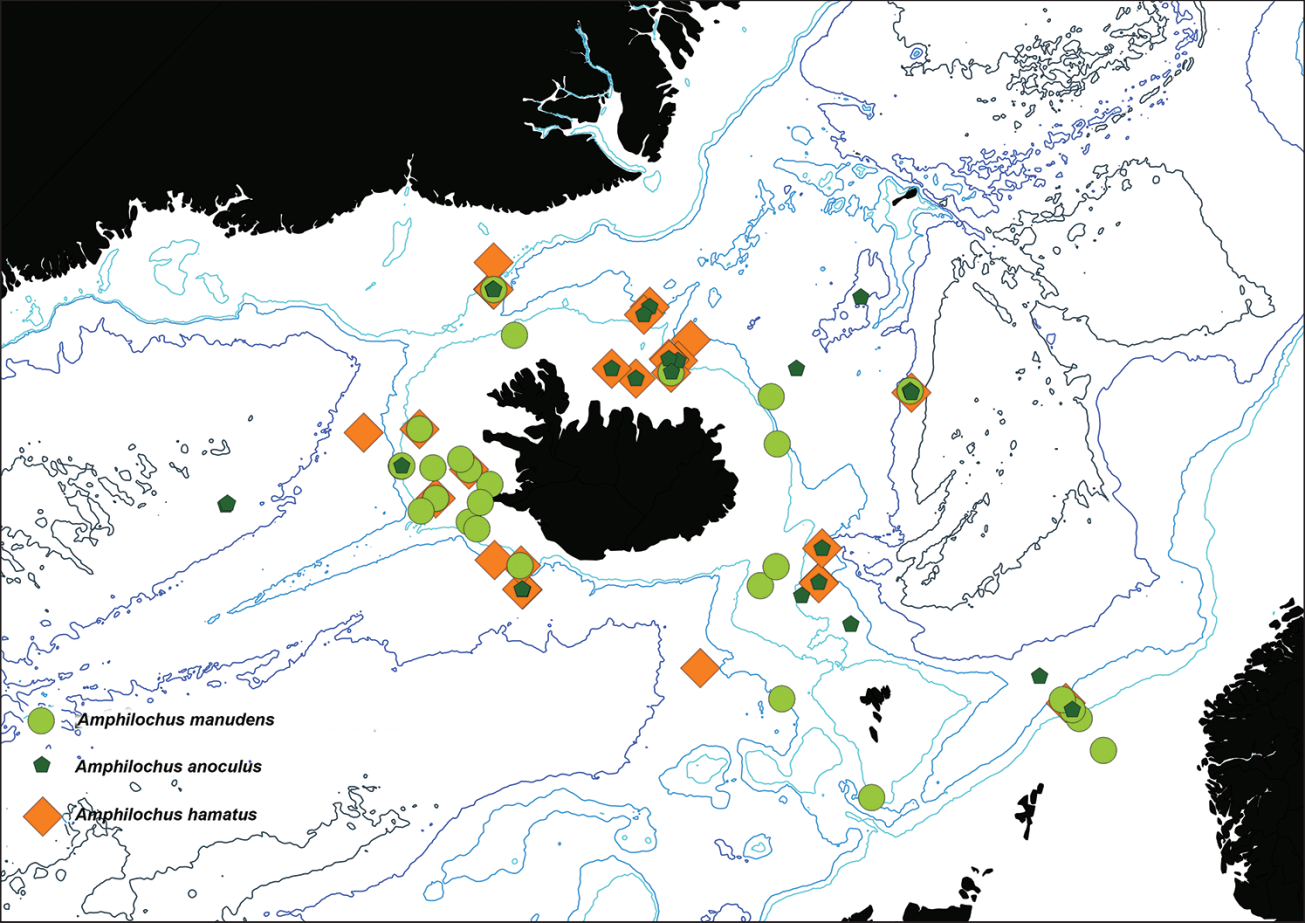
Map showing the Icelandic distribution of *Amphilochus anoculus* sp. n., *Amphilochus manudens* and *Amphilohus hamatus* (based on BioIce and IceAGE material).

This species is known from BioIce/IceAGE stations in deep and cold waters north and east of Iceland, from deep stations in the Faroe-Shetland Channel, several deep stations north in the Norwegian Sea and from one deep station in the polar basin. It appears to be confined to cold and deep waters (see Fig. 1).

**Figure 2.**
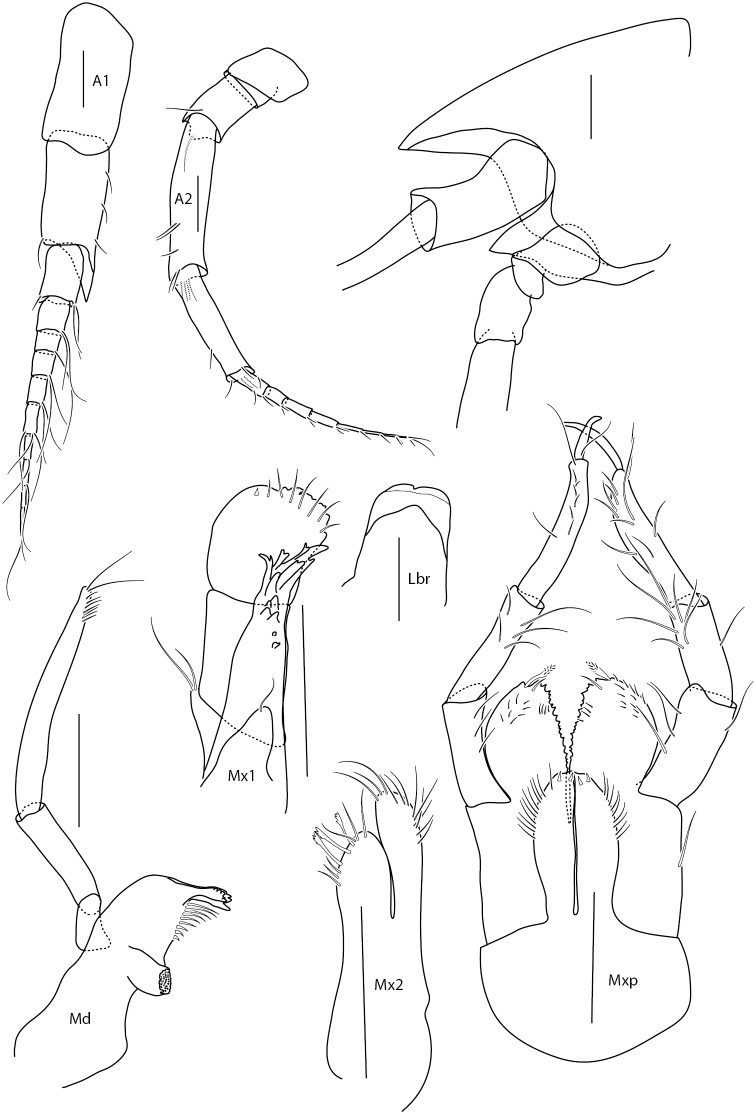
*Amphilochus anoculus* sp. n. Head and mouthparts. ZMBN121952. Scale bars: 0.1 mm.

**Figure 3.**
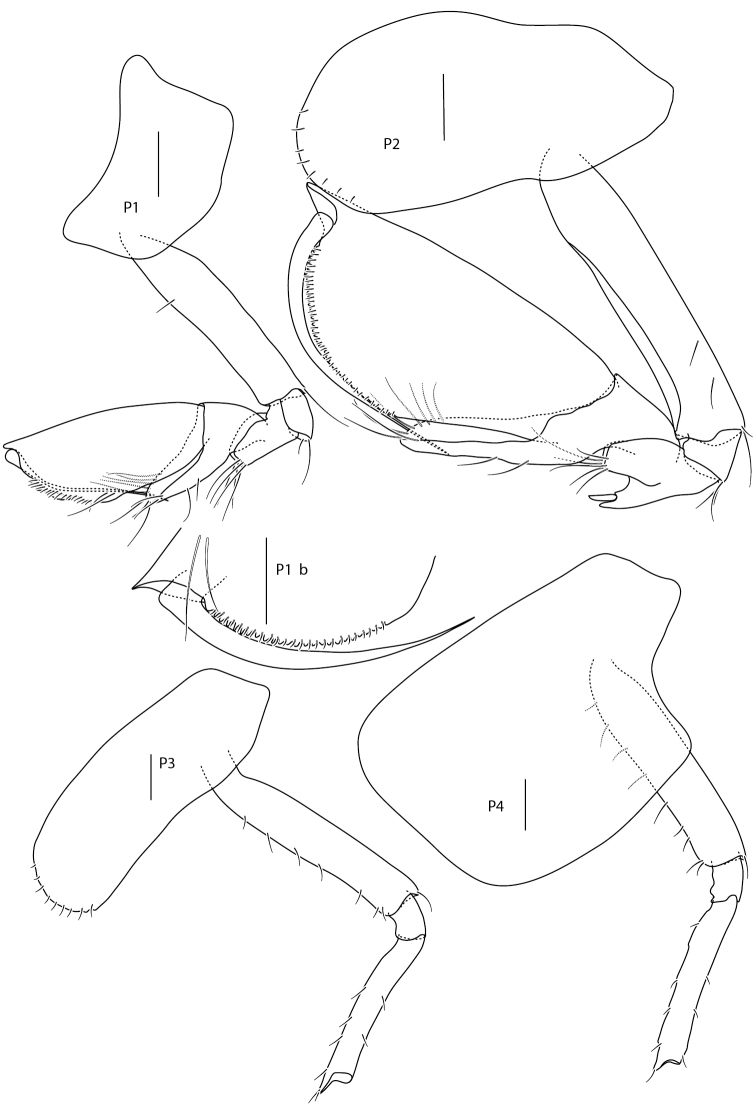
*Amphilochus anoculus* sp. n. Pereopods 1, 2, 3 and 4. ZMBN121952. Pereopod 1 dactylus from ZMH K-47225. Scale bars: 0.1 mm.

**Figure 4.**
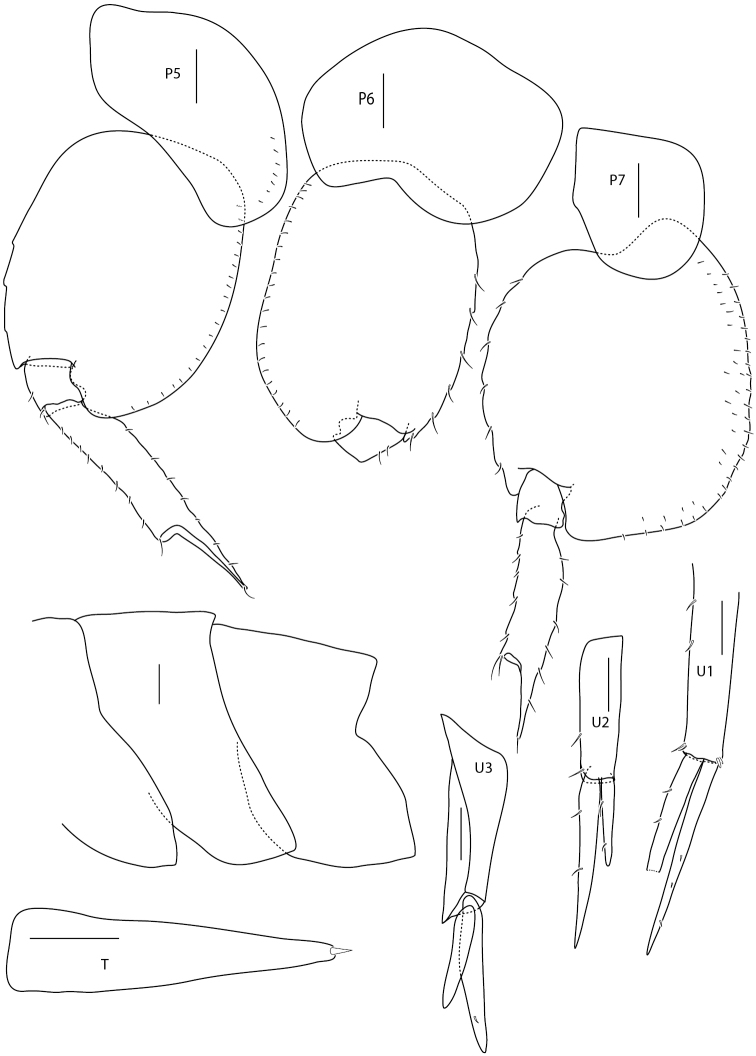
*Amphilochus anoculus* sp. n. Pereopods 5, 6 and 7, Epimeral plates, Uropods 1, 2 from ZMBN121952. Uropod 3 and telson from BioIce station 2367. Scale bars: 0.1 mm.

Illustrations are all from paratypes: Figs 2–4 of ZMBN121952, except for Fig. 3 pereopod 1 dactylus (1b) which is from ZMH K-47225 and Fig. 4 uropod 3 and telson that are both from BioIce station 2367.

######### Description.

Description is based on a composite of studied material. No observed sexual dimorphism.

*Head.* Rostrum subequal to peduncle article 1 of antenna 1, curved. Eyes absent. Cephalic lobes produced, broadly rounded, tips of mouthparts just visible under the edge of cephalon. Antenna 1 subequal to antenna 2; peduncle strong, longer than six-articulate flagellum; accessory flagellum absent. Setae on both peduncle and flagellum few and short. Antenna 2 peduncle longer than eight-articulate flagellum. Few and short setae distally on peduncle articles, all articles of the peduncle are longer than broad.

Labrum symmetrically bilobed. Mandible molar small but triturative, rounded cone-shaped, with setation on entire chewing area, which is ridged; incisor serrate; eleven accessory spines; palp slender, 3-articulate; article 1 is shorter than article 2, which is shorter than article 3; article 3 with setae; lacinia mobilis laterally expanded. Labium symmetrical, without inner lobes. Maxilla 1 palp biarticulate, with two apical setae; inner plate reduced, with one seta; outer plate with eight robust and six thinner setae. Maxilla 2 inner plate shorter than outer plate, nine setae on distal margin; outer plate long and thin with four distal setae. Maxilliped inner plate reaching end of merus, well separated, thin, two robust distal setae; outer plate reaches middle of carpus of palp, one robust seta and ridge of serrations; palp slim, heavily setulated on propodus.

*Mesosome* dorsally smooth; segment 3 is shorter than segment 4. Coxa 1 reduced and covered by coxa 2, which is longer than broad. Coxa 2 distal margin serrate and with setae. Coxa 3 and 4 distal margin not serrate, without setae. Coxa 5–7 concave.

Pereopod 1 basis longer than propodus, upper half distally widened, few and short setae; carpal lobe well developed, reaching 65% of posterior margin of propodus; propodus triangular; palm oblique, serrate with setae, no seta defining palmar corner, anterodistal tooth of medium size (half as long as the base of dactylus is broad); dactylus smooth with few, thin setae on inner margin. Pereopod 2 basis little longer than propodus, upper half not as widened distally as pereopod 1; carpal lobe covers all of posterior margin of propodus; propodus elongate, palm oblique, serrate with minute setae, no setae defining the palmar corner, anterodistal tooth well developed (same size as the breadth of the base of dactylus); dactylus inner margin weakly serrate on proximal half. Pereopod 3 missing in holotype. Pereopod 4 basis with four anterior setae, dactylus half-length of propodus. Pereopod 5 with posterior lobe on basis and merus. Pereopod 6 with posterior lobe on basis; posterior lobe on merus boat-shaped; carpus shorter than propodus; dactylus more than half length of propodus. Pereopod 7, posterior lobe on basis and merus, meral lobe covers 50% of carpus; dactylus more than half-length propodus.

*Metasome* smooth. Epimeral plates 1 and 3 rounded; plate 2 right-angled. Urosome smooth; segment 1 long; segments 2 and 3 shorter. Uropod 1 peduncle longer than rami; outer ramus marginally longer than inner; three to four setae on outer margins. Uropod 2 peduncle longer than rami; outer ramus half-length of inner; setae on both rami. Uropod 3 peduncle with clear flange, smooth; outer ramus weakly shorter than inner ramus; uropod 3 longer than telson. Gills on segments 2 to 7; oostegites on segments 2 to 6. Telson elongate and boat-shaped; distal end entire, acute and with one seta.

*Living colour*. Semi-transparent, virtually colourless.

######### Distribution.

Iceland, Faroe Channel, Norwegian Sea, Polar basin. Has only been found in cold and deep water.

######### Remarks.

This species is easily recognized because it lacks eyes and has an anterodistal tooth on the propodi of pereopods 1 and 2. *Amphilochus manudens* and *A. hamatus* are the only other Amphilochidae having this tooth, but unlike *Amphilochus anoculus* sp. n., they both have eyes. The telson has a robust seta distally, a character not seen in any other Amphilochidae. The flange on the distal end of uropod 3 peduncle is also a good character-state to use when separating it from *A. hamatus*. A synoptic list of characters separating the three species is shown in Table 2.

**Table 2.** Comparison of character states between *Amphilochus anoculus* sp. n., *A. manudens*, and *A. hamatus*.

**Character**	***Amphilochus anoculus* sp. n.**	***Amphilochus manudens***	***Amphilochus hamatus***
**Cephalic lobes**	rounded	acute	rounded
**Labrum**	symmetrically bilobed	asymmetrically bilobed	asymmetrically bilobed
**Mandible**	molar rounded	molar conical	molar conical
**1^st^ Maxilla**	palp 2-articulate	palp 2-articulate	palp 1-articulate
**2^nd^ Maxilla**	outer plate with 4 setae	outer plate with 3 setae	outer plate with 3 setae
**Labium**	tooth on inner edge of outer plate	no tooth on inner edge of outer plate	tooth on inner edge of outer plate
**Eyes**	absent	round, strongly coloured	ill defined, bean-shaped or oval
**Gnathopod 2**	elongate	subtriangular	elongate
**Oostegites on P6**	present	absent	present
**Epimeral plate 1**	rounded	angular	angular
**Epimeral plate 3**	rounded	with clear tooth	rounded
**Uropod 3**	with flange on peduncle	no flange on peduncle	no flange on peduncle
**Telson**	tip with robust seta	tip smooth	tip tridentate – all lobes rounded.
**Temperature (°C)**	-0.6 to + 1.7	+1.7 to + 7	-0.85 to +7
**Depth (m)**	303m to 1055m	81m to 350m (single specimens at 772m and 1390m)	260m to 1407m

######### Biology.

This species appears to be restricted to cold water (it is only found at a temperature range of -0.6 °C to +1.7 °C. Three stations from the Mareano-project have higher temperatures than this (stations R1180 RP86, R1200 RP90 and R1225 RP112). These are also the three of the shallowest stations where this species has been found, and constitute a statistical outlier in the dataset. They are all in the eastern Barents Sea, an area where winter-temperatures are much colder, and thus still might fall within the proposed ecological niche of the species. It has been found north and east of Iceland, south of the Faroe Islands, north in the Norwegian Sea and in the Polar basin, at depths ranging from 303 to 2100 meters. In contrast, the closely related *Amphilochus manudens* has, during BioIce, IceAGE, and several other collection efforts in the area been found mainly at depths from 81 to 360 meters, with single specimens found at 772 and 1390 meters (see Fig. 1 for specimens from BioIce and IceAGE). No *Amphilochus manudens* were found in the Faroe-samples from AkvaplanNiva.

######### Derivatio nominis.

The name *anoculus* (*an* = no, *oculus* = eye) refers to the absence of eyes. It is a noun in apposition.

######## Amphilochus manudens

Taxon classificationAnimaliaAmphipodaAmphilochidae

Spence Bate, 1862

Amphilochus manudens Spence Bate, 1862:107, pl 17 fig 6; Sars 1890-95: 217, pl 74; Chevreux and Fage 1925: 114, fig 109; Lincoln 1979: 150, fig 65 e-f, fig 66 a-d; Krapp-Schickel 1982: 75, fig 51.

######### Remarks.

Although *Amphilochus manudens* is one of the best described species within the Amphilochidae (Sars 1890–95; Lincoln 1979; KrappSchickel 1982), we have included a redescription of material from Iceland, to facilitate direct comparison with the new species.

######### Material examined.

**Figure 5.**
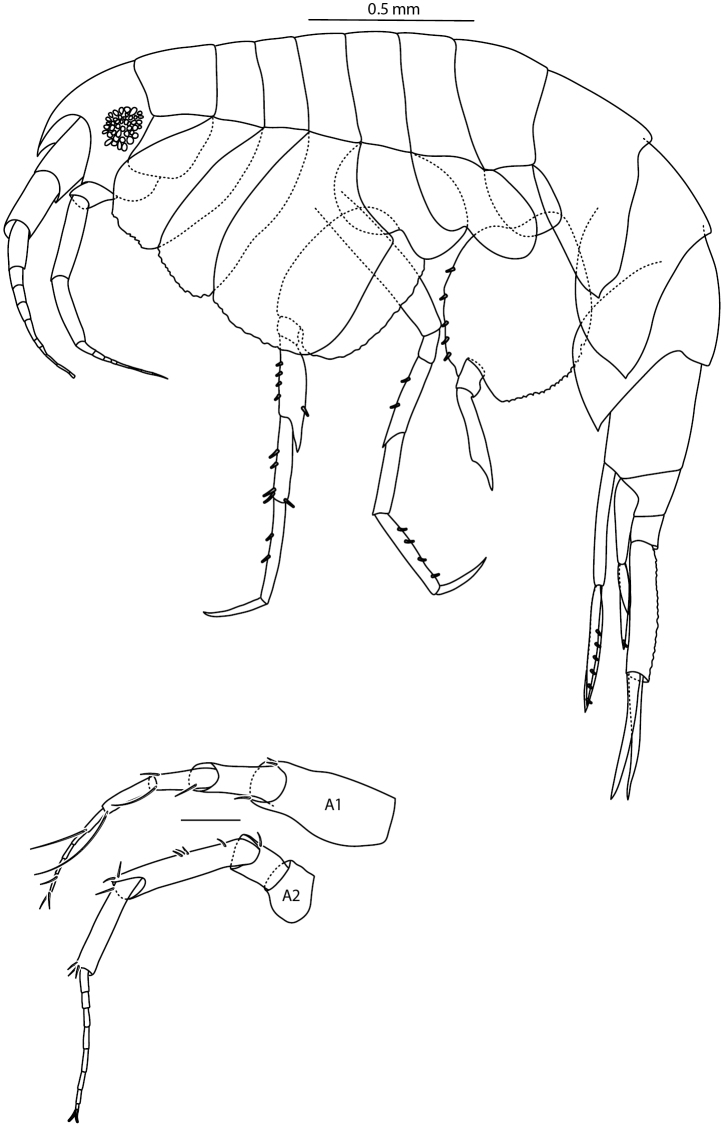
*Amphilochus manudens*. Habitus and antennae. IINH37889. Scale bar habitus 0.5 mm, other scale bars 0.1 mm.

**Figure 6.**
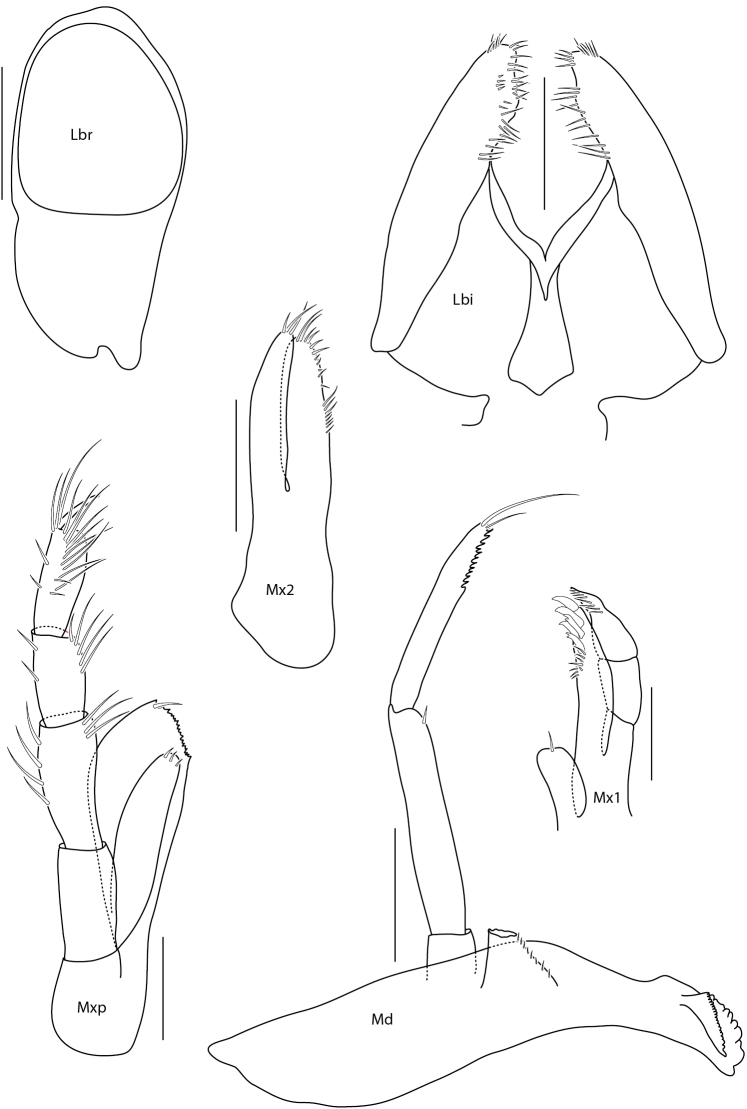
*Amphilochus manudens*. Mouthparts. IINH37887. Scale bars: 0.1 mm.

**Figure 7.**
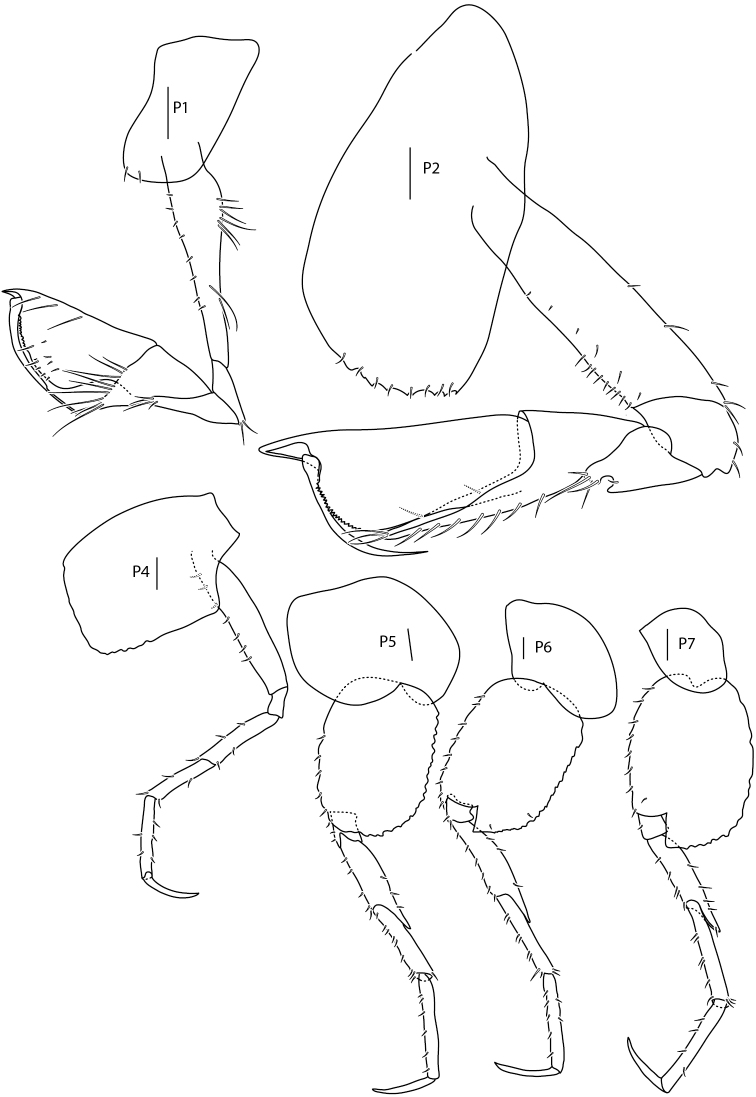
*Amphilochus manudens*. Pereopods. IINH37885. Scale bars: 0.1 mm.

**Figure 8.**
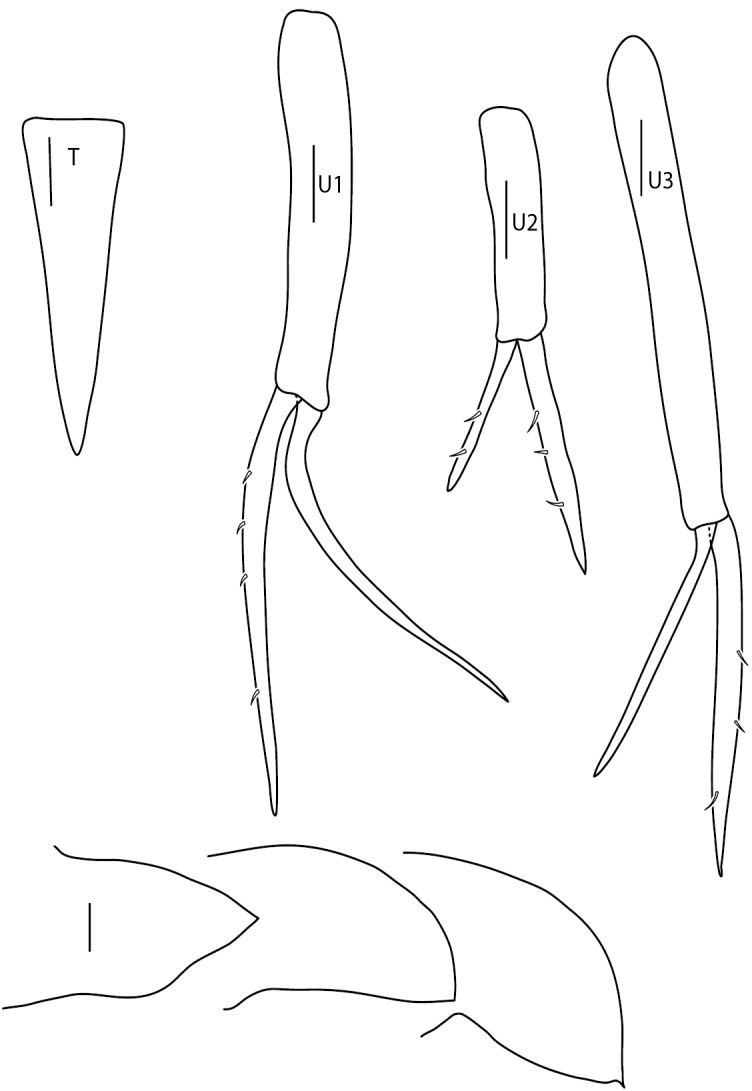
*Amphilochus manudens*. Appendages from pleon and urosome. IINH37885. Scale bars: 0.1 mm.

all drawings are made from specimens found during the BioIce program. For the complete set of drawings (Figs 5–8) we have used specimens IINH37889 (BioIce 2207), IINH37887 (BioIce 2215) and IINH37885 (BioIce 2237). Type material not examined. Additional material of *Amphilochidae* from a Statoil funded baseline survey of some Faroe waters has been examined, and only *Amphilochus anoculus* and *Amphilochus tenuimanus* were found. We have also examined all Amphilochidae from the BioFar program, and only *Amphilochus manudens* was found (no *Amphilochus anoculus* sp. n.). During a cruise in the Polar basin in 2005 both *Amphilochus manudens* and *Amphilochus anoculus* sp. n. were found, but at different stations (see discussion below). Material from several Norwegian surveys (summarised in the project NorAmph) and the IceAGE project included several *Amphilochus manudens*. For information about the specific sample-stations, see Table 1.

######### Description.

*Head.* Rostrum curved, smaller than peduncle article 1 of antenna 1. Eyes round, no ommatidial framing, small, deep brown-red in colour. Cephalic lobe produced, distally acute. Antenna 1 subequal to antenna 2; peduncle article 1 is longer than article 2, which is longer than article 3; peduncle is longer than six-articulate flagellum; accessory flagellum absent. Antenna 2 peduncle longer than eight-articulate flagellum; peduncle articles have few short setae.

Labrum asymmetrically bilobed. Mandible molar small but triturative, cone-shaped, with a row of short setae around the ridged chewing area; incisor serrate; nine accessory spines; palp slender, 3-articulate; article 1 is shorter than article 2, which is longer than article 3; article 3 with two long setae distally and distal third of margin serrate; lacinia mobilis laterally expanded. Labium symmetric; inner lobes reduced. Maxilla 1 palp 2-articulate, with eight setae; inner plate reduced, with 1 seta; outer plate with six strong setae and two rows with four and three smaller setae. Maxilla 2 inner plate shorter than outer plate, six long setae distally and a row of five short, three long and five short setae; outer plate is long and thin with three distal setae. Maxilliped inner plate is long and thin, well separated, three short and strong setae distally; outer plate reaches just past merus of palp; palp slim, heavily setulated on carpus and propodus.

*Mesosome* dorsally smooth; length segment 3 is smaller than segment 4. Coxa 1 reduced and covered by coxa 2, which is longer than broad. Coxa 2 distal margin serrate, with setae. Coxa 3 concave; distal margin serrate, without setae. Coxa 4 distal margin serrate; without setae. Coxa 5–7 concave.

Pereopod 1 basis longer than propodus, upper half distally widened, few and short setae on anterior margin, longer setae on posterior margin; carpal lobe well developed, 50% of posterior margin of propodus; propodus subtriangular, proximal half of oblique palm serrate, distal half with short evenly spaced setae, no seta defining palm, anterodistal tooth strong; dactylus longer than palm, narrow and acute, apparently smooth. Pereopod 2 basis longer than propodus, linear, several short setae; one robust seta distally on ischium; merus with small distal ‘hook’; carpal lobe covers 100% of posterior margin of propodus, lined with setae posteriorly, small crown of setae distally; propodus elongate with a regularly convex serrate palm without seta, anterodistal tooth strong; dactylus longer than palm, narrow, apparently smooth. Pereopod 3 coxa elongate, pereopod 4 coxa posteriorly produced, both with basis to propodus anterior edge lined with short setae, dactylus more than half propodus. Pereopod 5 to 7 basis and merus with posterior lobes; carpus shorter than propodus; dactylus longer than half propodus.

*Metasome* smooth. Epimeral plate 1 with small, blunt posterodistal tooth, distal margin convex; plate 2 angular, distal margin convex; plate 3 with clear posterodistal tooth, distal margin weakly concave. Urosome smooth; segment 1 as long as segments 2 and 3 together. Uropod 1 peduncle and rami subequal; rami subequal; setae on outer ramus. Uropod 2 peduncle subequal to inner ramus; outer ramus about half-length of inner ramus; setation on both rami. Uropod 3 peduncle longer than rami; outer ramus is shorter than inner ramus; rami longer than telson; rami with setae.

Gills on segments 2 to 6. Oostegites on segments 2 to 5. Telson elongate; distal end entire and acute; no setae.

######### Distribution.

North East Atlantic and Arctic Ocean (Lincoln, 1979); Barents Sea and Murmansk area (Gurjanova 1951; Vader and Bryazgin 1998; Vader et al. 2001); Spitsbergen (Stephensen 1935; Vader et al. 2001); Mediterranean (Marseilles, Capri) (Krapp-Schickel 1982); amphi-Atlantic (Watling 1979); Gulf of St Lawrence (Brunel et al. 1998).

######## Amphilochus hamatus

Taxon classificationAnimaliaAmphipodaAmphilochidae

(Stephensen, 1925) comb. n.

Amphilochopsis hamatus Stephensen, 1925: 173, figs 52–53; Gurjanova 1951: 402, fig. 246; Barnard and Karaman 1991: 95.

######### Material examined.

**Figure 9.**
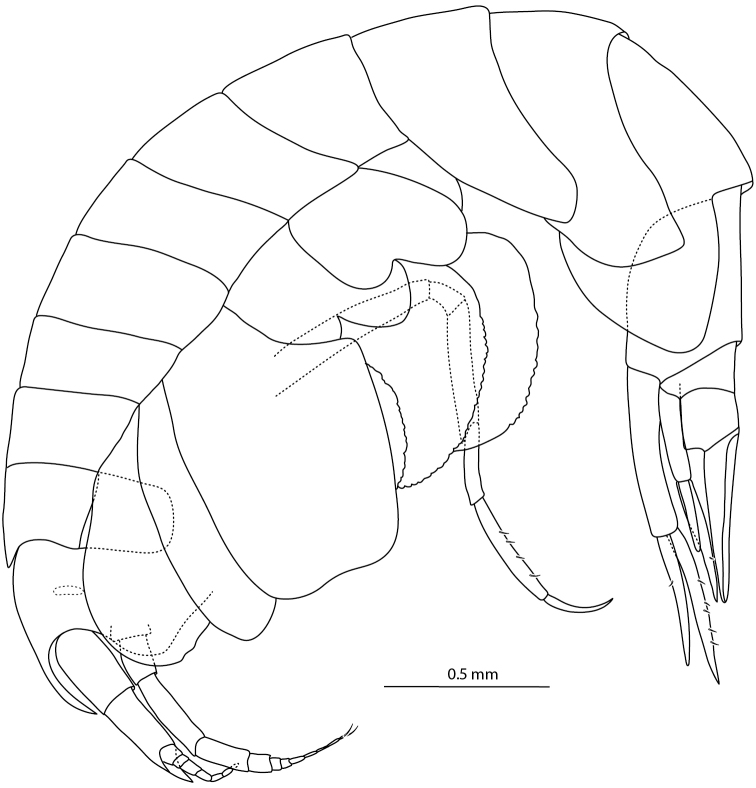
*Amphilochus hamatus*. Habitus. IINH37900. Scale bar: 0.5 mm.

**Figure 10.**
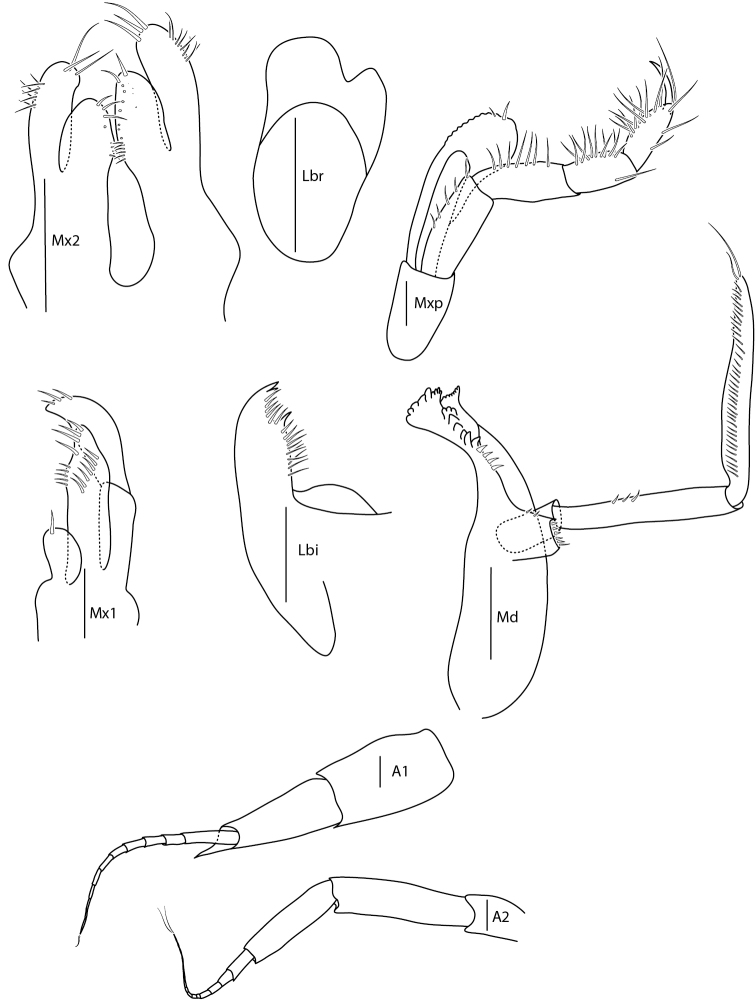
*Amphilochus hamatus*. Mouthparts. IINH37894. Scale bars: 0.1 mm.

**Figure 11.**
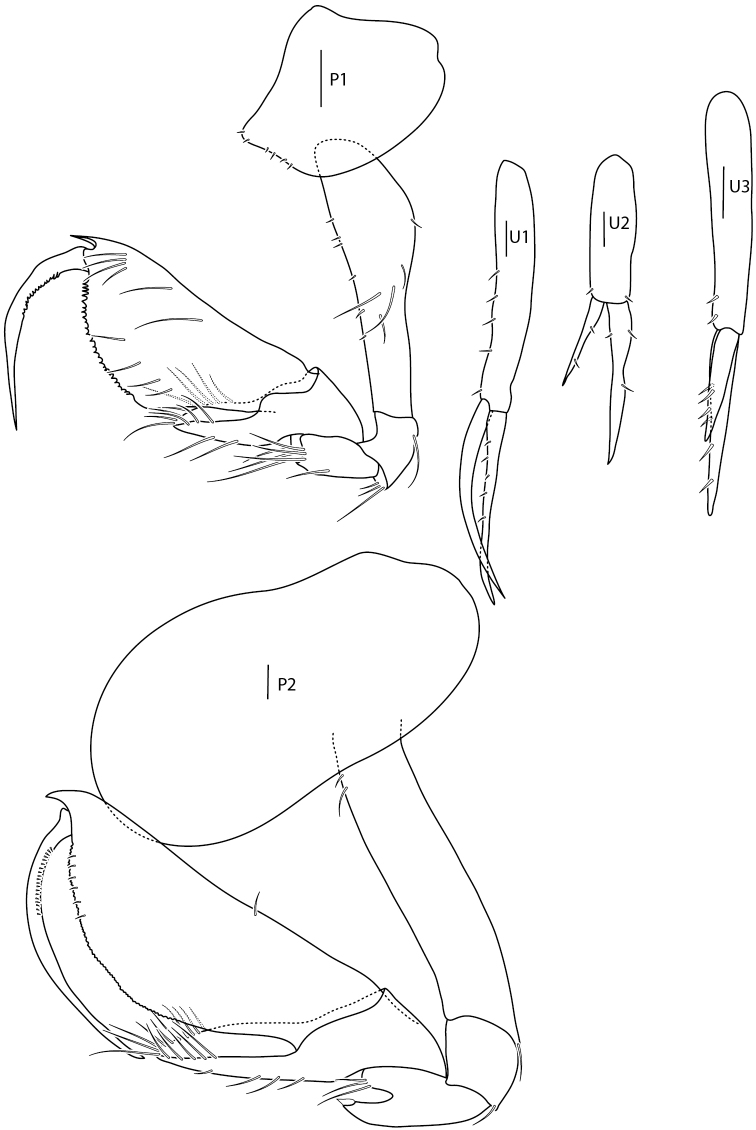
*Amphilochus hamatus*. Pereopods 1 and 2, uropods. IINH37898 and IINH37903. Scale bars: 0.1 mm.

**Figure 12.**
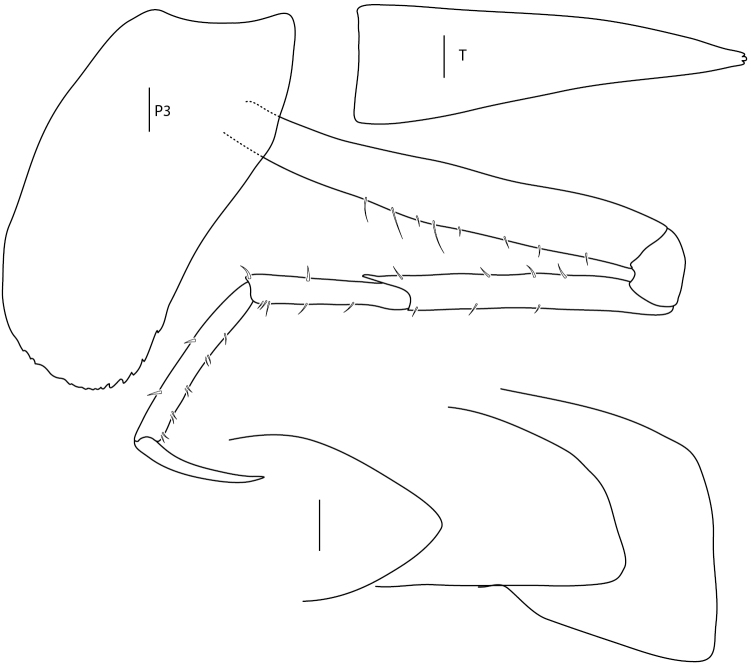
*Amphilochus hamatus*. Pereopod 3, epimeral plates, telson. IINH37898 and IINH37894. Scale bars: 0.1 mm.

Drawings are made from IINH37894 (BioIce 2077), IINH37898 (BioIce 2236), IINH37900 (BioIce 2318) and IINH37903 (BioIce 2367). Material from IceAGE and NorAmph has been used for molecular sequencing and comparisons. For a list of stations for the material, see Table 1. Type material not examined. The drawings are shown on Figs 9–12.

######### Description.

*Head*. Rostrum curved, reaches tip of article 1, antenna 1. Eyes not evident, but an ill-defined eye-patch can be seen. Cephalic lobe produced distally and rounded. Antenna 1 shorter than antenna 2; second peduncle-article with a triangular production on the apex the size of third peduncle article; peduncle subequal to ten-articulate flagellum; no accessory flagellum. Antenna 2 peduncle longer than flagellum; short setae on peduncle, and a pair of long setae at tip of flagellum.

Labrum asymmetrically bilobed. Mandible molar small but triturative, cone-shaped; incisor serrate; ten accessory spines; palp slender, 3-articulate with series of short setae on article 3, one long seta at tip; lacinia mobilis laterally expanded. Labium symmetric, with inner lobe reduced; sharp tooth making tip of outer lobe look dentate. Maxilla 1 palp 1-articulate, with a crown of two robust setae and a serrate distal margin; inner plate reduced, 1 seta; outer plate with four and eight heavy and five smaller setae. Maxilla 2 long and thin; inner plate shorter than outer plate; three heavy setae and eight smaller setae on outer plate; inner plate with seven short setae distally and one to two thin setae medially. Maxilliped inner plate small and thin, well separated, just reaching past ischium; outer plate reaching mid-merus, two strong setae distally, serrations on inner margin; palp slim, heavily setulated on carpus and propodus.

*Mesosome* dorsally smooth; segment 3 shorter than segment 4. Coxa 1 reduced, subquadratic and covered by coxa 2, which is longer than broad. Coxa 2 distal margin smooth, no setae. Coxa 3 concave, smooth, with setae on distal margin. Coxa 4 distal margin smooth with setae. Coxa 5–7 concave.

Pereopod 1 basis subequal to propodus length, upper half distally widened, three long setae posteriorly; carpal lobe 65% of propodus posterior margin; propodus subtriangular, palm oblique, serrate, no setae defining palmer corner, anterodistal tooth of medium size; dactylus with inner margin partly serrate. Pereopod 2 basis weakly longer than propodus, linear; merus with a clearly defined “hook” on posterior side, close to carpus; carpal lobe 100% of propodus posterior margin, boat-shaped with a row of setae on margin; propodus subovate, palm oblique, defined mostly by its serration and upper third with small setae, no setae defining palmar corner, anterodistal tooth large (same size as breadth of the base of dactylus); dactylus inner margin with a row of small and strong setae, otherwise smooth. Pereopod 3 posterior margin of basis with an even row of slender setae; merus with small lobe. Pereopod 4 basis with very thin setae. Pereopods 5 and 6 basis and merus with posterior lobes; dactylus longer than half-length propodus. Pereopod 7, basis and merus with posterior lobes.

*Metasome* smooth. Epimeral plates rounded, no teeth. Urosome smooth; segment 1 long; segments 2 and 3 short. Uropod 1 peduncle subequal to rami; rami subequal; both peduncle and outer ramus with marginal setae. Uropod 2 peduncle subequal to inner ramus; outer ramus half-length of inner ramus; setation on peduncle and rami. Uropod 3 peduncle marginally longer than rami; outer ramus half-length of inner ramus; rami shorter than telson; setation on peduncle and rami. Gills on segments 2 to 6. Oostegites on segments 2 to 6. Telson elongate, longer than broad; distal end entire and tridentate, all lobes rounded; no setae.

######### Distribution.

This species appears to have a wide depth range based on our collections (206 to 1407 m), although Stephensen (1925) found it only in deep water (700 to 2702 m). The temperatures it has been found at range from -0.6 to +7.0 °C. It is also recorded from the deep Norwegian Sea (Dahl 1979), the Arctic basin (Gurjanova 1951), Greenland (Brandt et al. 1996) and the deep polar basin (Tzvetkova and Golikov 2001).

## Discussion

### Genetic delimitation of the species

**Figure 13.**
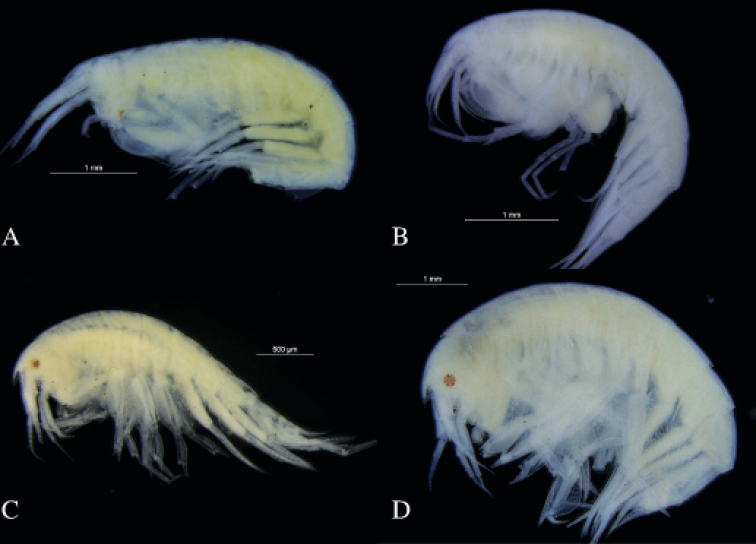
Photographs of habitus **A** *Amphilochus anoculus* sp. n. ZMBN_104532 **B** *Amphilochus hamatus* ZMBN_104479 **C** *Amphilochus manudens* ZMBN_103989 **D** *Amphilochus manudens* UMBergen_NBamph_123.

Examinations of the COI-gene (Folmer segment) of *Amphilochus manudens*, *Amphilochus anoculus* sp. n. and *Amphilochopsis hamatus* from both IceAGE (Icelandic waters) and NorBol (Norwegian waters) show a clear separation of the new species *Amphilochus anoculus* from other Amphilochidae tested. (Jazdzewska et al. 2018; NorAmph in Barcode of Life Project (BOLD) www.boldsystems.org). Using Barcode Identification Numbers (BIN) to make a quick check on species delimitation gives four different BINs for *Amphilochus manudens* from the two projects, as well as separate BINs for *Amphilochus anoculus* and *Amphilochopsis hamatus*. It has, however, been very difficult to get good sequences for *A. anoculus*; after thorough scrutiny we only found one non-ambiguous sequence. Many of our discarded sequences were removed from the analyses from being too short, but the parts we have are identical to the full COI-sequence we tested, and that thoroughly separates it from all clades of *A. manudens* and *A. hamatus.* Calculating the distance between groups using Mega7 (Kumar et al. 2015) shows this (Table 3), even though it must be noted that since *A. manudens* separated into several clades, the within-distance for this group was also very large (0.283). Clearly, a more thorough genetic analysis and possibly a larger sample-pool (especially a larger genetic sample pool) will reveal if we have further new species to be separated from *Amphilochus manudens*, but for this study it will suffice to note that *Amphilochus manudens* may constitute a species complex. Specimens of *Amphilochus manudens* assigned to two of the different BINs as well as *Amphilochus anoculus* sp. n. and *Amphilochopsis hamatus* are photographed (Fig. 13).

**Table 3.** P-distances between groups (species) of Amphilochidae from NorAmph and IceAGE projects.

*Amphilochoides boecki*	0,426					
*Amphilochus anoculus*	0,343	0,359				
*Amphilochus hamatus*	0,331	0,302	0,196			
*Amphilochus manudens*	0,388	0,400	0,335	0,279		
*Amphilochus sp1*	0,336	0,317	0,223	0,095	0,238	
*Amphilochus tenuimanus*	0,357	0,327	0,312	0,285	0,378	0,294

### The status of the genus *Amphilochopsis* Stephensen, 1925

The genus *Amphilochopsis* was erected by Stephensen (1925) for the species *A. hamatus*. Stephensen wrote: ‘The present genus is very closely allied to *Amphilochus*, but is characterised especially in having the molar of the maxillae (*sic*!) well developed (but not very large) and in having only one joint in the palp of maxilla 1’.The type species of the relatively large (Barnard and Karaman 1991) and probably not monophyletic (Hoover and Bousfield 2001) genus *Amphilochus Spence* Bate, 1862 is *Amphilochus manudens Spence* Bate, 1862; this species is usually described as having a non-triturative molar on the mandible, but in reality the molar, although much reduced in size and conical in form, has a small flat triturating surface on top (see Fig. 6). *Amphilochus manudens* has a 2-articulate palp on maxilla 1. Later authors have often allied the genus *Amphilochopsis* with the basic amphilochid genus, the also extremely variable *Gitanopsis* G.O. Sars. This is probably mainly because in keys to the genera the first dichotomy usually concerns the molar, and *Amphilochopsis* is deemed to have a well-developed molar, while *Amphilochus* is judged to have a feebly developed, non-triturative molar. Thus Barnard and Karaman (1991) write in their monograph for *Amphilochopsis* sub ‘Relationship’: ‘Differing from *Gitanopsis* in the 1-articulate palp of maxilla 1’, and also Hoover and Bousfield (2001), in their phenograms, ally *Amphilochopsis* closely to *Gitanopsis*.

In reality the molar of *Amphilochus manudens* is only quantitatively different from that of *Amphilochopsis hamatus*, with the new species *A. anoculus* in an intermediate position between the two. These molars are completely different from the well-developed cylindrical molars of *Gitanopsis* and *Gitana* species, as well as from the almost completely reduced molars of many other species in *Amphilochus* s.l. A number of other species in *Amphilochus* s. l., e.g. the west Atlantic *A. casahoya* and *A. delacaya*, both described by McKinney (1978), and the Hawaiian species described by Barnard in 1970, have the same type of ‘intermediate’ molar as *A. manudens*.

*Amphilochopsis hamatus* has a clearly 1-articulate palp on mx 1, while all *Amphilochus* species that we have seen have a 2-articulate palp. This type of character-state has been used extensively elsewhere in the division of genera in the Amphilochidae (cf. the discussion in Barnard (1962)). We feel, however, that this difference alone is not sufficient to warrant a separate genus for *A. hamatus*, especially as the articulation of the palp in some *Amphilochus* species, i.e., *A. anoculus*, is not always easy to perceive and may even be incomplete.

As shown by Hoover and Bousfield (2001) who in their ‘partial revision’ split up *Amphilochus* s. l. and erected the genus *Apolochus* for some of its species, *Amphilochus* s. l. is definitely not a monophyletic genus, and is in great need of a complete revision. A preliminary phylogenetic analysis of amphilochid species, based on literature data (Tandberg 2000) came to the same conclusion: species of *Amphilochus* and *Gitanopsis* were scattered over the entire cladogram. The cladogram did, however, show a clear clade around *Amphilochus manudens*, the type species of *Amphilochus*, and thus *Amphilochus* s. str.: this clade included, besides *A. manudens*, the new species *A. anoculus*, *A. opunake* Barnard, 1972 from New Zealand, the Mediterranean *A. planierensis* Ledoyer, 1977, and *Amphilochopsis hamatus*.

An easily observed and spectacular character of *A. hamatus* is the characteristic hook on the merus of P2, from which its name is derived. However, this same hook occurs, albeit in greatly reduced form, in both *A. manudens* and *A. planierensis*. The new species described above, *A. anoculus*, also has a meral hook on P2; this is another character where the character state present in *A. anoculus* falls between the more extreme versions of the states in *A. manudens* and *A. hamatus*. We therefore do not think the meral hook on P2 to be of more than specific value.

For these reasons, we have decided to transfer *Amphilochopsis hamatus* to *Amphilochus* s. str. and to submerge the genus *Amphilochopsis* as a junior synonym of *Amphilochus* s. str.

### Ecology of the species

In Icelandic waters, *Amphilochus manudens* and *Amphilochus hamatus* seem to be confined to shallower and warmer waters. The only parameter that seems to be limiting is temperature – they are only found in “warm” waters: + 1,7˚C to +7˚C. *Amphilochus manudens* is common, and from the literature known to be found mostly on gravel and silty sand, and on hydroids (Jones 1948; Schellenberg 1942).

Given the distribution-data on *Amphilochus manudens* from BioIce, BioFar, IceAGE and other studies in the Faroe channel and our surveys in the Norwegian Sea and Polar basin, it seems that *Amphilochus anoculus* sp. n. replaces *Amphilochus manudens* in cold waters. Temperatures for the stations in the Faroe channel and a few in the Norwegian Sea were not reported, but Westerberg (1990) has shown that the general benthic temperatures in this area are always lower than 0.5 °C and temperatures in the deep waters of the Norwegian Sea are lower than 1 °C, which supports our hypothesis that *A. anoculus* replaces *A. manudens* at temperatures below 1.7 °C.

### Key to *Amphilochidae* in the North-East Atlantic

**Figure 14.**
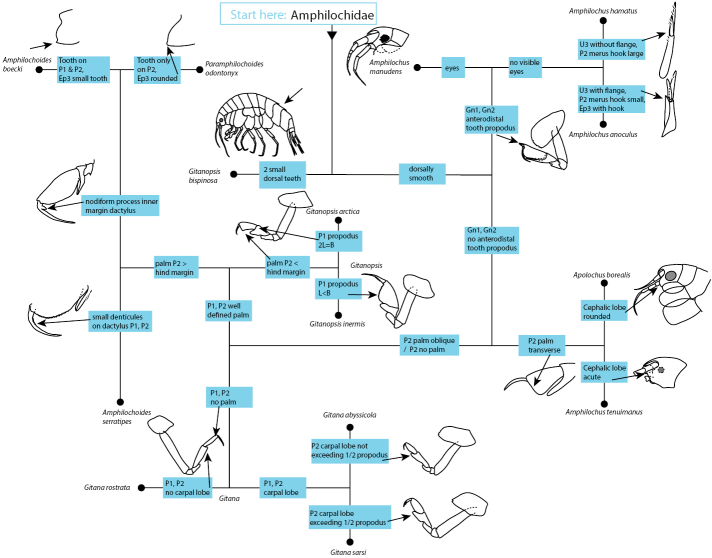
Pictorial key to Amphilochidae in the NE Atlantic.

A pictorial key, loosely based on Stephensen (1935) with the new species added, is shown in Fig. 14.

## Supplementary Material

XML Treatment for Amphilochus anoculus

XML Treatment for Amphilochus manudens

XML Treatment for Amphilochus hamatus
